# Polarization-sensitive optical coherence tomography with a conical beam scan for the investigation of birefringence and collagen alignment in the human cervix

**DOI:** 10.1364/BOE.10.004190

**Published:** 2019-07-24

**Authors:** Wei Li, Brenda F. Narice, Dilly O. Anumba, Stephen J. Matcher

**Affiliations:** 1 Biophotonics Group, Department of Electronic and Electrical Engineering, University of Sheffield, Sheffield, S3 7HQ, UK; 2 Reproductive and Developmental Medicine, Department of Oncology and Metabolism, University of Sheffield, Sheffield, S10 2SF, UK; 3 Co-first authors with equal contribution.

## Abstract

By measuring the phase retardance of a cervical extracellular matrix, our in-house polarization-sensitive optical coherence tomography (PS-OCT) was shown to be capable of (1) mapping the distribution of collagen fibers in the non-gravid cervix, (2) accurately determining birefringence, and (3) measuring the distinctive depolarization of the cervical tissue. A conical beam scan strategy was also employed to explore the 3D orientation of the collagen fibers in the cervix by interrogating the samples with an incident light at 45° and successive azimuthal rotations of 0-360°. Our results confirmed previous observations by X-ray diffraction, suggesting that in the non-gravid human cervix collagen fibers adjacent to the endocervical canal and in the outermost areas tend to arrange in a longitudinal fashion whereas in the middle area they are oriented circumferentially. PS-OCT can assess the microstructure of the human cervical collagen in vitro and holds the potential to help us better understand cervical remodeling prior to birth pending the development of an *in vivo* probe.

## 1. Introduction

Preterm birth (PTB), which is defined as birth before 37 weeks of gestation, is the leading cause of neonatal morbidity and mortality not attributable to congenital malformations worldwide. It accounts for more than 1 million deaths a year [1]. Across the world, more than 15 million births are preterm every year, with prevalence rates that range from 5% to 18% [2–4]. In the UK, around 8% of babies are born prematurely whereas in the US approximately 12% of live births occur before term [5,6]. Despite advances in perinatal health, the incidence of PTB has continued to increase. Given the multifactorial etiology of PTB, diagnosis and prevention have proven difficult. However regardless of what triggers PTB, there seems to be common gradual changes in the stroma of the cervix. The cervix, which plays an essential part in maintaining a pregnancy to term, has to remain closed throughout gestation so that the fetus can develop in utero [7]. However, for birth to occur, it has to shorten, soften and dilate. This crucial remodeling process is required for uterine contractions to lead to delivery [8,9]. Since PTB requires premature cervical remodeling, improved understanding of this process is essential for the development of more accurate screening tools for PTB [10]. Such tools may also facilitate better targeted clinical interventions. Cervical remodeling begins several weeks/months before parturition, but its exact timing and processes have not yet been fully characterized in humans, most evidence stemming from studies on rodents [11]. Experiments conducted on rat and human cervical biopsy tissues in the late 1980s using X-ray diffraction showed that collagen fibrils exhibited preferential orientation in the non-pregnant cervix: around the endocervical canal and in the outermost area, collagen fibers were mostly arranged longitudinally, whereas in the middle area, fibers were predominantly circumferential [12]. This orientation pattern is thought to be lost during pregnancy. Current evidence also suggests that cervical remodeling involves a change in the orientation, morphology and assembly rather than in collagen amount. However, current *in vivo* assessment of the remodeling of the cervix in women is confined to cervical length ultrasound measurement and digital examination approaches incapable of assessing the key molecular changes associated with extracellular matrix remodeling [9].

Several research imaging techniques have been employed to investigate cervical collagen microstructure, including X-ray diffraction, second harmonic generation (SHG) microscopy, magnetic resonance diffusion tensor imaging (MR DTI) and optical coherence tomography (OCT) [13–17]. However, none of these modalities has been successfully translated into the clinical setting due to inherent limitations to the technique. MR DTI, for example, is too slow for real-time processing; SHG holds limited imaging speed and does not perform well endoscopically, and OCT lacks accuracy to assess the collagen structure. An emerging technique, Full-field Mueller colposcopy, has also been developed for investigation of cervical microstructure [18–21]. This technique has regarded as a potential alternative to the current screening methods, e.g. histological diagnoses, due to the advantages of low cost, rapid imaging with wide field images and ready endoscope [19]. However, the technique of Full-field Mueller colposcopy is not in the mainstream clinic yet, and cannot provide depth-resolved changes in tissue’s phase retardance, birefringence and relative fast axis orientation nor the thickness of the overlying epithelium.

Polarization-sensitive OCT (PS-OCT) is a functional extension of OCT, which has the potential to be an appropriate tool for investigation of cervix or cervical remodeling in clinical studies. This is because PS-OCT not only shares the advantages of OCT, including high resolution (4-20 μm), high-speed 3-D imaging and easy integration with a catheter or a hand-held probe, but it offers additional information such as the polarization state of backscattered optical light [22,23]. The polarization state can be used to measure tissue’s depth-resolved phase retardance, birefringence and relative fast axis orientation, which allows PS-OCT to differentiate anisotropic tissues, such as collagen fiber, muscle and tendon, from other structures [24]. In 2008, Lee et al demonstrated that PS-OCT could detect cervical intraepithelial cancer (CIN) on human cervical biopsies with a sensitivity of 94.7% and a specificity of 71.2% when results were correlated with histology [25]. However, little is known about the ability of PS-OCT to assess changes in the orientation of cervical collagen [26].

In this study, we sought to assess whether PS-OCT was capable of detecting changes in the alignment of cervical collagen fibers in vitro. Cervical cross-sections obtained from uterine specimens of patients undergoing hysterectomy for benign gynaecological conditions were fully scanned with PS-OCT. Additionally, the three-dimension (3D) orientation of collagen in the samples was assessed using a conical beam scan protocol, originally developed for studying collagen alignment in articular cartilage [27].

## 2. Methods

### 2.1 Configuration of PS-OCT

**Fig. 1:**
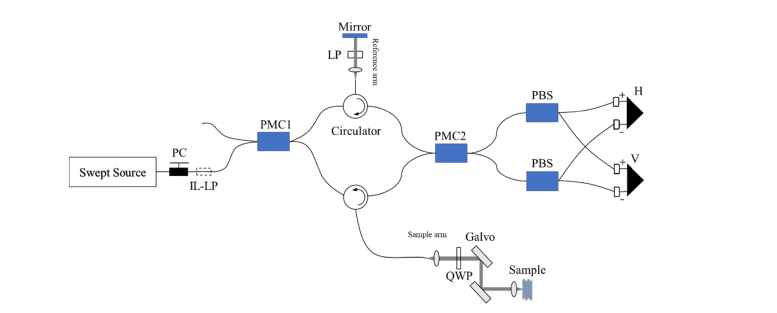
Schematic diagram of PS-OCT system, where PC is polarization controller, IL-LP is in-line linear polarizer, PMC is polarization maintaining coupler, QWP is quarter waveplate, PBS is polarization beamsplitter and H and V are balanced photo-detectors used to detect horizontally and vertically polarized optical signals, respectively This diagram is modified from [27].

Our in-house PS-OCT for this study was developed based on the method reported by Al-Qaisi et al [28]. This system and its characteristics have already been described in our previous paper [27]. Here, we provide concise summaries of the PS-OCT configuration and its principle. The schematic diagram of PS-OCT is shown in Fig. 1

. The light source of the system was a wavelength-swept laser (HSL-2000-10-MDL, Santec, Japan) with a center wavelength of 1315 nm, a full width at half maximum of 128 nm, a wavelength scanning rate of 10 kHz and a duty cycle of 60%, which supplied an output power of 10 mW to system. The light emitted from the light source firstly passed through a polarization controller (PC) and an in-line linear polarizer (IL-LP) to yield linearly polarized light. Then, the polarized light entered a polarization-maintaining fiber (PMF) based Mach-Zehnder interferometer. In the Mach-Zehnder interferometer, the light was split into 10% of light for reference arm and 90% of light for sample arm by a 2 × 2 polarization-maintaining coupler (PMC1, OLCPLP-22-131-10-90-FA, Opto-link Corp., China). The PMC1 was aligned with the transmission axis of light to allow the light to be only coupled into the slow axis of PMF. The two beams were then directed into the reference and sample arms via two three-port polarization-maintaining circulators (OLCIR-P-3-131-300-90-FA, Opto-link Corp., China) respectively. In the reference arm, the light transited through a linear polarizer (LP) oriented at 45° to the slow axis of PMF, and was reflected back to circulator by a static plane mirror. As a result, the reference light subsequently entering into the detection arm had a polarizing angle of 45° with respect to the slow axis of PMF and equal intensity components in the horizontal and vertical directions of the slow axis of PMF. The light in the sample arm firstly travelled through a quarter wave plate (QWP, NT55-547, Edmund Optics, US) oriented at 45° to the slow axis of PMF, which produced a circularly polarized light for interrogation of sample. Then, the circularly polarized light transited through a galvanometer system (6215, Cambridge Technology, US) and a Thorlabs LSM03 OCT scanning lens (f = 36mm) sequentially, and was scattered by a sample. The galvanometer system consisting of a pair of galvanometer scanners was used to achieve functions of B-scan and volumetric scan. The scanning lens was used to focus the light and generate a light spot with diameter of 25 μm in the focal plane. The light backscattered by the sample carrying sample’s information was recombined and interfered with the light from the reference arm at another 2 × 2 polarization-maintaining coupler (PMC2, OLCPL-P-22-131-50-90-FA, Opto-link Corp., China). PMC2 was equally split the light into two polarization beamsplitters (PBS, PBS-31-P-2-L-3-Q, NovaWave Techno., US). The PBS divided the combined light into horizontally and vertically polarized light. Two balanced detectors (1817-FC, New Focus, US) were used to detect the horizontally and vertically polarized lights respectively. The light signals were sampled by a 14-bit transient recorder (M2i.4022, Spectrum GmbH, Germany) at 20 MS/s for measuring and processing the interferences of horizontally and vertically polarized lights. Axial resolution of ~10μm in air has been characterized by an optical mirror in this PS-OCT system.

### 2.2 Preparation of human cervical tissues

The study was granted approval from the Yorkshire & Humber Committee of the UK National Research Ethics Service (REC Number 08/H1310/35) and the University of Sheffield (Registration number 160268520). Written consent was sought and obtained from all participants. Twenty non-gravid cervical samples were obtained from women undergoing vaginal, total abdominal or laparoscopic-assisted hysterectomy for benign conditions such as menorrhagia, prolapse or endometriosis. Clinical and demographic data including age, parity, previous mode of delivery, body mass index (BMI), indication for surgery, type of surgery, menopausal status and hormonal treatment was anonymized and coded for statistical analysis.

**Fig. 2:**
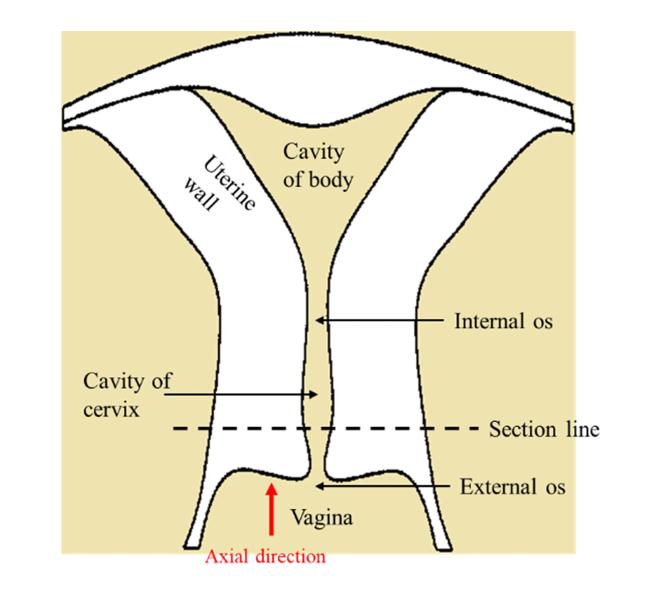
The diagram of uterus. The specimen was prepared by cutting along the section line. The red arrow denotes the axial direction of imaging in our experiments.

Cervical cross-sections approximately 20 mm thick were excised from the uterine specimen by a single operator, as illustrated shown in Fig. 2

, and soon after incubated in 0.1 mg/ml streptomycin, 0.25 µg/ml amphotericin B and 100 U/ml penicillin, and stored at −20°C on Human Tissue Authority (HTA) licensed premises until processing with PS-OCT. For PS-OCT scanning, the specimens were thawed in phosphate-buffered saline (PBS) at room temperature, and then sealed in an optical-window cell culture dish for imaging.

### 2.3 Scan geometries

**Fig. 3:**
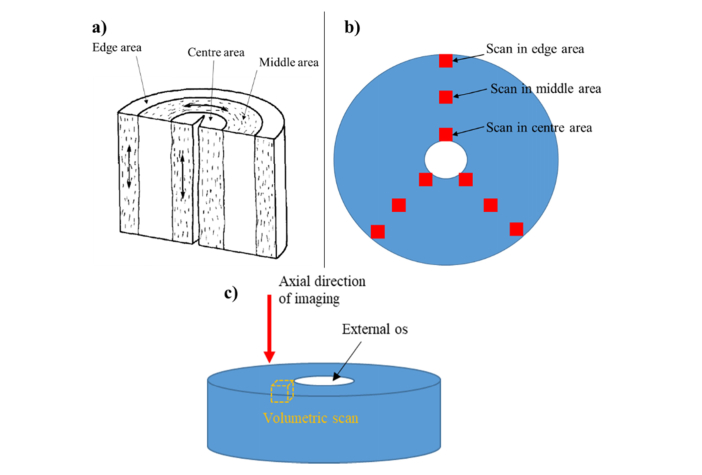
a) Schematic diagram of a human cervix, modified from Aspden (1988) [12]. The preferential orientation of collagen fibrils is shown as short dash in the three distinct cervical areas, named as center, middle, and edge areas respectively. b) Top view of cervical specimen with 9 scanning points. c) Axial direction of imaging within the cervical sample from the external to the internal os.

Each specimen of cervical tissue was imaged using two different PS-OCT techniques. In the first protocol, a volumetric scan was achieved by combination of 1000 b-scans to produce a 3D image with x y z size of 4 by 4 by ~2.15 mm. The volumetric scan was repeated 9 times at different points, as shown in Fig. 3(b)

. These points were retrieved from three different areas, arbitrarily labelled as center area, middle area and edge area, consistent with the preferential orientation exhibited by the collagen fibers in the cervix as seen in Fig. 3(a) [12,16,29]. The axial direction of imaging was from external os to internal os and perpendicular to surface of sample, shown in Fig. 3(c).

**Fig. 4:**
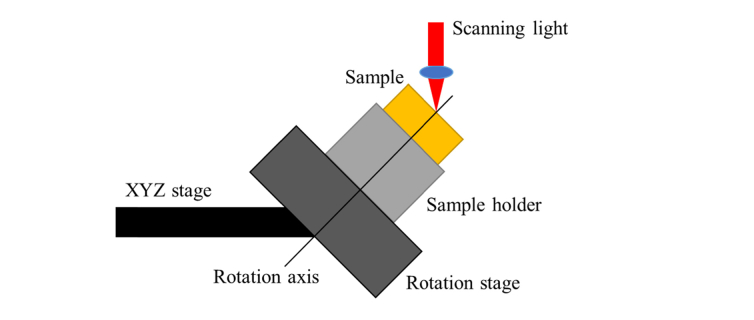
The schematic diagram of sample stage for conical beam scan scheme. The incident angle of scanning light is 45°. The incident light strikes the point where the rotation axis intersects sample surface. The specimen is imaged by successive B-scans with a synchronized motorized rotation stage, which obtain 360 B-scans spanning azimuthal angle of 1°-360° with an interval of 1°.

The second scanning technique, which was used to evaluate the 3D orientation of cervical collagen, entailed the use of the so-called conical beam scan strategy previously described by our research group in the 3D characterization of collagen fibers in articular cartilage [27]. A schematic diagram of the conical beam scan is shown in Fig. 4

. A motorized rotation stage was synchronized to the A-scan data acquisition and mounted on a manual XYZ stage for position adjustment. A sample holder mounted on the rotation stage was then used to fix sample and fine tune position. The scanning light of PS-OCT struck the point where the rotation axis intersected sample surface at a 45° incidence angle. The sample was imaged by acquiring successive B-scans with synchronized stage rotation. The successive B-scans were acquired every 1° as the motorized stage rotated the sample over 360°. In the 360 B-scans, the A-scans which had the same height of sample surface at the rotation axis were selected and plotted as a polar format, where the radial distance indicated the axial imaging depth. The polar format was then used to evaluate the 3D orientation of the cervical collagen as described in our reported method [27].

### 2.4 PS-OCT image processing

Our image processing was carried out in MATLAB. The retardance $\left(\delta_{S}(z)\right)$ image was calculated as $\delta_{S}(z)=\arctan(A_{0;V}(z)/A_{0;H}(z)),$ where $A_{0;V}(z)$ and $A_{0;H}(z)$ indicate the amplitudes of the vertical and horizontal signals respectively. The apparent birefringence of the specimen (Δn), which refers to the refractive index difference between the specimen ((n) and the ordinary beams ($(n_{o}),$ namely $\Delta n=n-n_{o},$ was computed as:

$$\Delta n=\frac{\lambda_{0}}{2\pi }\frac{d\delta_{S}(z)}{dz}$$ (1)

where z is the physical depth into sample and $\lambda_{0}$ is the center wavelength of the light source. The true birefringence, defined as $n_{e}-n_{o}$, can be expressed as following relationship [30]:

$$\Delta n=n_{o}\left(\frac{n_{e}}{\sqrt{{n_{o}}^{2}+\cos^{2}(\theta_{C})({n_{e}}^{2}-{n_{o}}^{2})}}-1\right)$$ (2)

Here, $n_{e}$ is the extraordinary refractive index and $\theta_{C}$ is angle between the direction of light propagation and the optic axis of the fiber, i.e. the c-axis in optical terminology. For obtaining more precise values of the apparent birefringence, the phase retardances of 10000 A-scans (arranged in an XY grid of size 100 by 100) at each depth within the sample were averaged, and plotted as a function of depth to get the retardance slope of the birefringent tissue, e.g. $d\delta_{S}(z)/dz$. The slope was obtained by linear fitting method, and the birefringence values were calculated as illustrated in the Eq. (1). To visualize apparent birefringence, a 2D birefringence image was mapped out by the derivative of the retardance versus axial image depth after the retardance B-scan was smoothed using a 50 by 50 median filter.

The depolarization of tissue was quantified on the basis of the theory developed by Götzinger et al [31]. The degree of polarization uniformity (DOPU), which can be regarded as a spatially averaged degree of polarization (DOP), was quantified for evaluation of tissue depolarization. DOPU was processed as follows. Firstly, a thresholding procedure was applied to the intensity data (I), i.e. $I=A_{0;V}{\left(z\right)}^{2}+A_{0;H}{\left(z\right)}^{2},$ for filtering out noise and low signal intensity. Secondly, the Stokes vector (*S*) was computed as [31]:

$$S=\left(\begin{array}{c} I\\ Q\\ U\\ V \end{array}\right)=\left(\begin{array}{c} A_{0;V}{\left(z\right)}^{2}+A_{0;H}{\left(z\right)}^{2}\\ A_{0;V}{\left(z\right)}^{2}-A_{0;H}{\left(z\right)}^{2}\\ 2A_{0;V}(z)A_{0;H}(z)\cos\Delta \phi \\ 2A_{0;V}(z)A_{0;H}(z)\sin\Delta \phi \end{array}\right)$$ (3)

where *I*, *Q*, *U*, and *V* are Stokes vector elements, and then the Strokes vector elements were averaged by a 2D mean filter (a size of 15 by 6 pixels). Finally, the DOPU was processed in term of $DOPU=\sqrt{{Q_{m}}^{2}+{U_{m}}^{2}+{V_{m}}^{2}},$ where $Q_{m},$ $U_{m}$ and $V_{m}$ denote the averaged Stokes vector elements.

### 2.5 H&E histology

Three cervical specimens, previously scanned with PS-OCT were then fixed with 3.7% formaldehyde and stained with a modified H&E technique in order to better visualize collagen fibers. Histological slides were subsequently assessed with an optical microscope (10X, LEICA DM750).

### 2.6 Statistical analysis

Collagen birefringence in the center, middle and edge areas was compared using ANOVA with Bonferroni correction. Collagen birefringence was also correlated with age, parity, mode of delivery, menopausal status and indication for surgery using Pearson’s correlation coefficient, ANOVA when the assumption of equality of variances was met and non-parametric Kruskall-Wallis when the Levene’s test was significant.

## 3. Results and discussion

### 3.1 Intensity, retardance and birefringence images

**Fig. 5:**
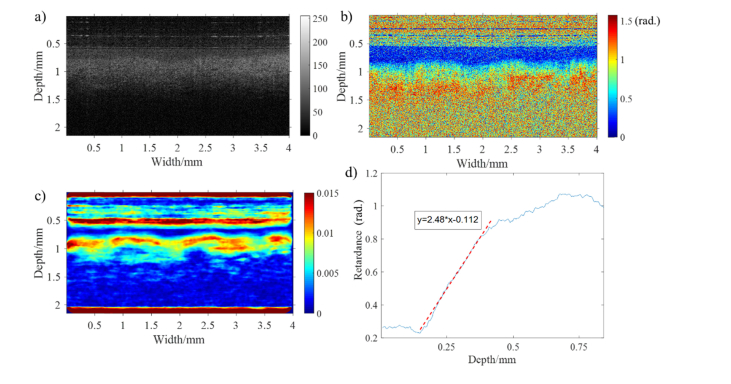
PS-OCT images of human cervical tissue in middle area: a) intensity image, b) retardance image, c) birefringence image (Note that the birefringence situated at sample surface layer is artifact, because the rapid change of retardance between noise and sample surface can lead to false birefringence signal.). d) the plot of averaged retardance as a function of depth (Red dashed: linear regression line of retardance for calculating birefringence of collagen).

Whole in vitro cervical cross-sections were scanned with our in-house PS-OCT. We have included, as an example, intensity, retardance and birefringence images obtained from the middle region of one of the samples analyzed, and shown how they were computed into precise numerical values (Fig. 5

). The intensity and retardance images were acquired using LABVIEW control software, shown in Figs. 5(a) and 5(b) respectively. In Fig. 5(a), the intensity image resulting from the difference of refractive index between various layers of tissue displays two discernible tissue layers. The superficial layer which presents comparatively lower intensity is assumed to be the cervical epithelium, and the deeper layer, the collagen content of the stroma. This assumption also enables to explain the retardance image, in which the cervical epithelium can be discerned as a blue band on the top due to its lack of birefringence. The thickness of the cervical epithelium can therefore be calculated immediately which could be of potential benefit in evaluating disorders such as cervical cancer [32] and the acquired human immunodeficiency syndrome [33]. Just underneath the epithelium layer, a significant increase of retardance is observed resulting from the birefringence of aligned collagen fibers. Compared with traditional modality, e.g. confocal fluorescence microscopy, which might cannot measure the epithelial thickness because the maximum imaging depth (<33 μm) is insufficient to cover all epithelial thickness [32], PS-OCT has much larger imaging depth (~800 μm) to readily measure the thickness of epithelium. In our experimental results, the retardance image has the advantage to differentiate the epithelial and collagen layers than intensity image, because a part of intensity images is featureless.

For generating a birefringence image, the gradient of retardance as a function of physical depth is computed after smoothing the retardance image using a median filter. The birefringence image is mapped out by the gradient of retardance, from which the collagen distribution can be inferred, shown in Fig. 5(c). The precise value of apparent birefringence of collagen is evaluated with a linear fitting method, shown in Fig. 5(d). In Fig. 5(d), the retardance at each particular depth within sample is laterally averaged to reduce speckle noise and plotted as a function of depth. The slope of collagen retardance, namely $d\delta_{S}(z)/dz,$ is calculated by linear regression. The precise value of birefringence can be directly calculated from Eq. (1). In our example, the slope of the regression equation in Fig. 5(d) is 2.48 rad/mm, and the value of collagen birefringence is $2.48/2\pi \times 1.315\times 10^{-3}\approx 0.52\times 10^{-3}.$ Since the retardance increases linearly with depth within sample, it is expected that the birefringence of collagen stays constant with depth. Background noise is gradually dominated at the deeper depth, masking the linear increase of retardance.

### 3.2 Orientation of collagen fiber

**Fig. 6:**
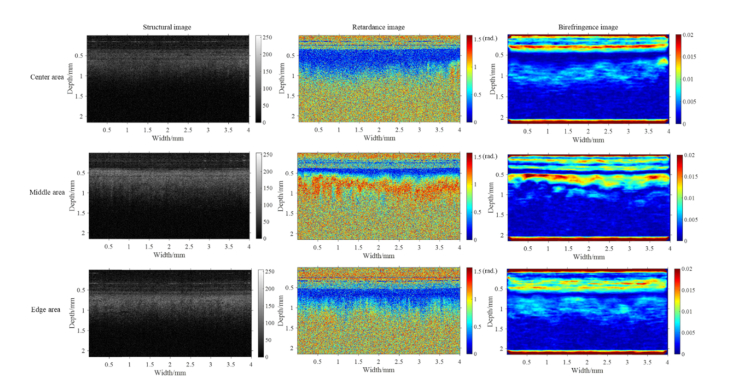
The structural, retardance and birefringence images of a cervical sample. Underneath sample surface, middle area has more evident birefringence and more significant increase of retardance as a function of depth than center and edge areas.

The cervical birefringence measured so far refers to the so-called apparent birefringence, which depends on imaging direction relative to orientation of the collagen fiber. In contrast, true birefringence, defined as $n_{e}-n_{o},$ is independent of imaging direction and fiber orientation, and can be regarded as an intrinsic value of the tissue. The relationship between apparent birefringence and true birefringence can be presented as illustrated in Eq. (2). According to this equation, the apparent birefringence reaches its maximum value when the light propagation is perpendicular to the c-axis of fiber, and becomes zero when the light travels along the c-axis. Therefore, we can roughly estimate the orientation of the c-axis by finding the imaging direction which yields minimal birefringence. Histologically, the collagen within the human cervix is thought to be aligned vertically in the edge and center areas, and circumferentially and horizontally in the middle area, shown in Fig. 3(a) [12,16,29]. Thus, in normal PS-OCT measurements in which the imaging direction is perpendicular to the sample surface, it is expected that the apparent birefringence (Δn) will reach its maximum value in the middle area whereas in the center and edge areas it will be close to 0. This expectation has been confirmed in our experimental results, shown in Fig. 6

. A set of b-scans obtained from one of the samples is displayed to exemplify how retardance and birefringence varies in the center, middle and edge areas in Fig. 6. In the middle area, retardance increases much more notably as a function of depth when compared with the center and edge areas. Consistently, the collagen birefringence in the middle area is higher and much more obvious than in the center and edge areas as seen in the birefringence images of Fig. 6, allowing differentiation between the different cervical regions. However, the difference of birefringence between the center, middle and edge areas is difficult to be identified through corresponding structural OCT images in Fig. 6.

A more accurate estimation of c-axis orientation has been realized by our group using variable incidence illumination directions to obtain a set of Δn and $\theta_{C}$ [30,34,35]. We assumed a value for $n_{o}$ and a positive uniaxial birefringent crystal structure for collagen fiber. The unknown polar orientation and extraordinary refractive index $n_{e}$ can be estimated by the simultaneous solution of a set of Eq. (2). However, this evaluation method was shown to be unsuitable for clinical application and *in vivo* measurements because it required controlling the illumination angle and solving the over-constrained problem to get $n_{e}$ and $\theta_{C}$. In order to tackle these difficulties, our research group developed an alternative conical beam scan technique capable of determining the 3-D orientation of the c-axis, and with potential for clinical endoscopic use [27]. This conical-beam method has the added benefit of modeling the variation of the c-axis versus depth if the sample has variable c-axis orientation in different layers [27]. Given these advantages, we have applied this specific conical-beam method to our cervical samples in order to assess the 3-D orientation of the c-axis.

**Fig. 7:**
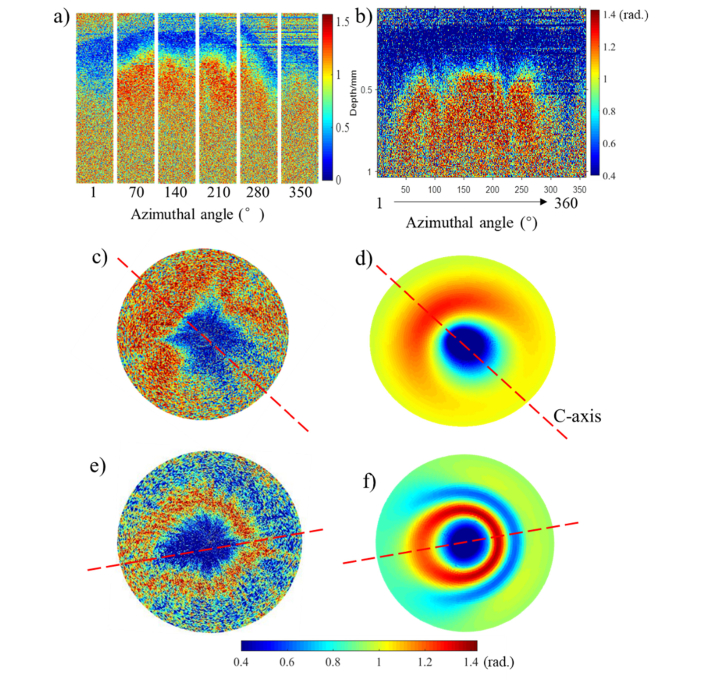
Real phase retardance images of human cervical tissue obtained by conical beam scan method and simulated retardance images using EJMC model. a): a series of B-scan retardance images in cervical center area acquired with a 45° incidence angle and a various of azimuthal angles. 360 A-scans of phase retardance extracted from the same data set are represented as a function of azimuthal angles in entire range of 1-360° with interval of 1° in conventional OCT image display format b) and polar format c). The corresponding simulated result is shown as d). e): 360 A-scans of phase retardance obtained in middle area represented as polar format. f): corresponding simulated result of e). Polar radius is 0.8 mm.

Using the conical beam, both the middle and center area of cervical samples were scanned. Following the conical beam scan proposal described in Section 2.3, 360 B-scans of the cervical sample were obtained with imaging directions of 45° incident polar angle and 1-360° azimuthal angles (with an interval of 1°) at each detecting point. The polar angle is defined as angle between the incident k-vector and normal direction of the sample surface. For the azimuthal angle, the sample surface is the reference plane, and the reference vector is the projected vector of initial light beam on the sample surface. Retardance images acquired by the conical beam scan in center area are shown in Fig. 7

. For a crude approximation of the collagen orientation, a series of B-scans at 1°, 70°, 140°, 210°, 280° and 350° azimuthal angles have been cropped and combined in Fig. 7(a). In Fig. 7(a), the B-scans at 70°, 140° and 210° azimuthal angles have a more evident increase in retardance as a function of depth, i.e. birefringence, than at other incident angles, contrary to what we would expect from our previous knowledge about cervical collagen arrangement, and what stems from Eq. (2). This is because according to the classic model of the human cervix, collagen fibers in the center area should be mainly arranged vertically [12]. As a consequence, the angle between the direction of light propagation and the c-axis, $\theta_{C}$, should remain unchanged during conical beam scan, and the birefringence obtained from all of azimuthal angles should be roughly equal in theory. To explain this discrepancy, we hypothesized that the c-axis of the collagen fiber is actually oriented at a polar angle tilted away from the normal axis. The polar and azimuthal angles of collagen fiber can be estimated by comparing simulated results of retardance images and real results in polar format. The estimation process and results are shown in Figs. 7(b), 7(c), and 7(d). 360 a-scans of phase retardance as a function of azimuthal angle are displayed in the traditional OCT format in Fig. 7(b), where each A-scan is fetched from each B-scan one by one over the azimuthal angle from 1° to 360°. These A-scans are extracted by a semi-automatic program to ensure every extracted A-scan corresponds to the center point of the plate rotation or close to the center point. This program runs on the assumption that the only center point of rotation has a constant altitude of sample surface in each B-scan due to the curved cervical surface. Therefore, the program is designed to find the 360 A-scans which have small variation of the surface altitude in each B-scan. The 360 A-scans are then converted to polar format, shown in Fig. 7(c), where the circle center and radius are sample surface and depth respectively. A simulated patterning of phase retardance in polar format is generated by a layered model based on the extended Jones Matrix calculus (EJMC) previously developed by our group [36], shown in Fig. 7(d).

The general process of EJMC is introduced briefly here. In the EJMC model, the sample of biological tissue is treated as a multi-layered structure, and each layer is considered as a linear retarder with a constant fast axis orientation. In this case, the signal-pass Jones matrix of sample (*P*) is the product of Jones matrices of individual layer, which can be expressed as:

$$P=\prod_{i=m}^{1}R(-\psi_{i})\left(\begin{array}{cc} e^{-i{k_{oz}}_{i}d_{i}} & 0\\ 0 & e^{-i{k_{ez}}_{i}d_{i}} \end{array}\right)R(\psi_{i})$$ (4)

Here, $R(\psi_{i})$ is the rotation matrix that diagonalizes the layer Jones matrix (i.e. defines the apparent fast-axis of the layer), $k_{oz_{i}}$ and $k_{ez_{i}}$ are the z component of the ordinary wave and extraordinary wave vectors, respectively, and $d_{i}$ is the thickness of ith layer. The details and formulas describing these terms (e.g. $R(\psi_{i}),$ $k_{oz_{i}}$ and $k_{ez_{i}}$) can be found in previous papers [37,38]. In brief, the Extended Jones Matrix Calculus of Gu and Yeh [38] is used to calculate $R(\psi_{i}),$ $k_{oz_{i}}$ and $k_{ez_{i}}$ from the true birefringence of each layer, $n_{e}-n_{o},$ and the polar and azimuthal angles of the layer c-axis and k-vector in the ith layer. Therefore, when we assume that the interface between the different layers has negligible specular reflection, the round-trip Jones Matrix of tissue $\left(J_{sample}\right)$ can be written as:

$$J_{sample}=T_{R}P^{T}PT_{R'}$$ (5)

where $T_{R}$ and $T_{R'}$ are the Fresnel reflection coefficients at the interface between air and sample surface. The light beam of PS-OCT (e.g. circularly polarized light) passing through individual layers of sample and then reflected back onto the detector can be modelled as:

$$\left(\begin{array}{c} A_{0;H}(z)\\ A_{0;V}(z) \end{array}\right)=R(45{}^{\circ})\cdot QWP\cdot R(-45{}^{\circ})\cdot J_{sample}\cdot \frac{1}{\sqrt{2}}\left(\begin{array}{c} 1\\ i \end{array}\right)$$ (6)

Here, *QWP* denotes the Jones Matrix of the quarter wave plate in PS-OCT system. Consequently, the depth dependent retardance $\left(\delta_{S}(z)\right)$ of sample detected by PS-OCT can be calculated as: $\delta_{S}(z)=\arctan(A_{0;V}(z)/A_{0;H}(z))$. The parameters of EJMC, including the ordinary refractive index, true birefringence and polar and azimuthal angles of collagen over the depth of the sample, can be set to find a simulated image which matches the pattern of the real image, and hence characterize 3D cervical collagen architecture. In our simulation, the sample has been divided into 40 layers over a total thickness of 1 mm.

The conical beam scans from the cervical center and middle areas are illustrated in Figs. 7(c) and 7(e), respectively, and their corresponding simulation images are shown in Figs. 7(d) and 7(f), respectively, in which the red dash represents the azimuth of c-axis. The simulation image of Fig. 7(d), for estimating orientation and properties of collagen in center area, is generated with the following parameters: zero birefringence over the depth of 0-100 μm (corresponding to the cervical epithelium), the true birefringence value of $2\times 10^{-3}$ over the depth of 100-500 μm, the ordinary refractive index of 1.37 over whole depth, the constant collagen polar angle of 10° over the depth of 100-500 μm and the constant azimuthal angle of collagen over the depth of 100-500 μm. Therefore, we estimated that the collagen fibers which we detected at that point in the center area were oriented at a polar angle of 10° tilted away from the normal axis. A similar approach was employed to assess the orientation of collagen fibers in the middle area as shown in Figs. 7(e) and 7(f). In this case, the depth of 0-200 μm had zero birefringence (corresponding to the thickness of cervical epithelium), and the collagen fibers had a fixed polar angle of 80° over the depth of 200-600 μm with true birefringence value of $1.3\times 10^{-3}.$ We concluded that the fibers in the middle area were not strictly arranged in a circumferential fashion, which has roughly horizontal arrangement with deviation. In keeping with a previous SHG study [17], our observations further challenge the classical model of collagen arrangement in the human cervix, and suggest the existence of a more complex structure.

### 3.3 H&E histology

**Fig. 8:**
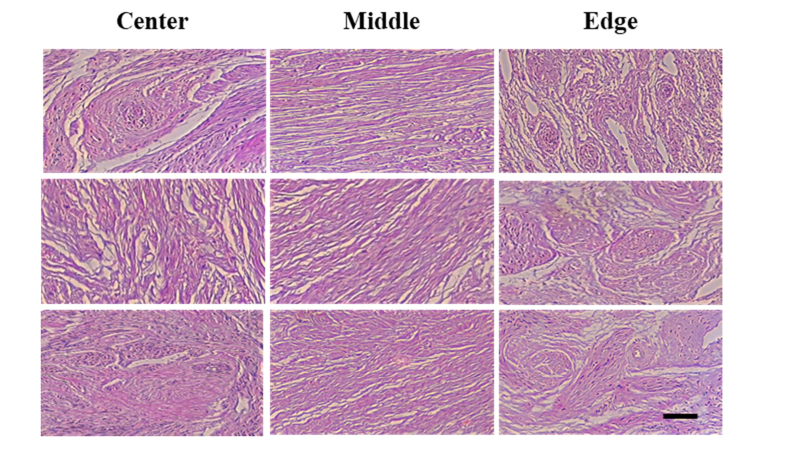
Histological slides of three cervical specimens. The left, central and right columns are the histological slides acquired from the center, middle and edge cervical area respectively (scale bar = 100 μm).

The histological slides of three cervical specimens which were previously scanned with PS-OCT are shown in Fig. 8

. When the histological slides were qualitatively reviewed, collagen fibers appeared more parallel aligned in the middle region, whereas in the edge and center areas, cross-sections of collagen bundles were more evident. Consistent with our PS-OCT findings, the analysis of the histological slides suggests that collagen fibers in the middle area are more circumferentially organized whereas those adjacent to the endocervical canal and in the outermost areas are mainly arranged in a longitudinal fashion. In addition, various degrees of collagen polar angle, near 80°, have been observed in the middle area, shown in the central column of Fig. 8, which confirms our conclusion obtained from conical beam scans.

### 3.4 Depolarization image

**Fig. 9:**
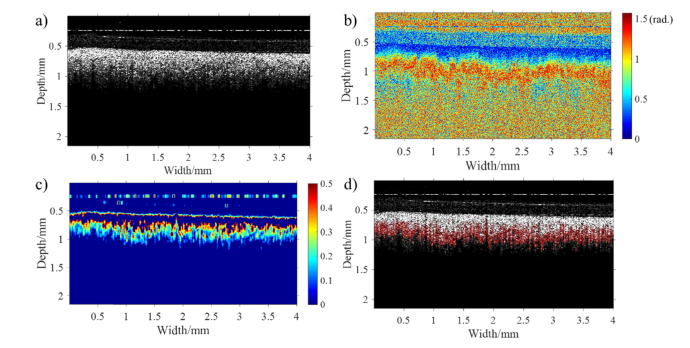
PS-OCT images of a human cervical specimen in meddle area for analyzing depolarization of tissue: a) Intensity image; b) retardance image; c) DOPU image displayed below a threshold of DOPU = 0.5; d) the overlay of structures with DOPU in red in the intensity image. The opacity of red indicates the degree of depolarization.

Polarization scrambling or loss of linear or circular polarization for tissue, termed as depolarization, can also be used in biomedical imaging to better discriminate between structures. PS-OCT is able to measure the depth-resolved depolarization of a sample using an equivalent parameter: DOPU [31]. Following the same computational algorithm as the one described in Section 2.4, the DOPU of human cervical tissue can be measured and visualized as shown in Fig. 9

. At this particular tissue site, the structural B-scan (Fig. 9(a)) shows negligible contrast between epithelium and stroma (c.f. the significantly better structural contrast seen in Fig. 5(a). In Fig. 9(c), the DOPU of cervical tissue in the middle area is normalized in the range of 0-1, and displayed below a threshold of DOPU = 0.5. Since the DOPU value indicates the correlation between the polarization state of speckle and adjacent speckles, the tissue prefers to preserve polarization if DOPU is close 1. Conversely, when the tissue has a high degree of depolarization, DOPU is near 0 [31]. The cervical epithelium has the DOPU value beyond the threshold, namely >0.5, in Fig. 9(c). Therefore, a polarization preserving layer can be seen for the cervical epithelium. In Fig. 9(d), the tissue depolarization is overlaid on the intensity image, and the opacity of red refers to the degree of depolarization. It is shown that the tissue with high depolarization corresponds to the collagen rich stromal layer which has obvious increase of retardance versus image depth in the retardance image (Fig. 9(b)) and high birefringence.

The polarization preserving character of a tissue has been reported to depend on the size and density of the scatters, the polarization states of illumination light, transport albedo, the sample birefringence, the diattenuation and the optical properties of the surrounding medium [39–42]. Several studies have concluded that (1) the depolarization of tissue has shown to be proportional to the number of scattering events and transport albedo, and (2) birefringent tissue normally displays a higher degree of depolarization than non-birefringent tissue. Consistently, our experiments have shown that birefringent tissue, i.e. collagen fibers, has higher level of depolarization than non-birefringent tissue, i.e. cervical epithelium. In preclinical applications, the depolarization assessed with PS-OCT has been used to explore and identify carious lesions [43] and retinal pigment epithelium [31,44–46] with promising results. DOPU has been shown to be an excellent indictor for detecting and quantifying natural pigment, such as melanin granules [24,44,45]. Thus, the tissue depolarization is another potential biomarker of PTB.

### 3.5 Benefits of PS-OCT for evaluating PTB

In summary, PS-OCT can directly measure a number of cervical parameters, including depth-resolved changes in intensity, phase retardance, birefringence, collagen orientation and depolarization. The depth-resolved information allows us obtain additional biomarker information, such as the epithelial thickness and 3D cervical collage architecture, which other modalities find difficult to provide. For example, Full-field Mueller colposcopy cannot measure the epithelial thickness and the out-of-plane fiber orientation, since it only gives us depth-wise average information. In addition, confocal fluorescence microscopy cannot provide equivalent information due to insufficient imaging depth as discussed in section 3.1. All these physical measurements could potentially become valuable biomarkers for evaluating PTB. However, to verify the feasibility of these measurements in clinical applications, statistically powered clinical trials of the different approaches and systematic reviews of the results are required.

### 3.6 Statistical analysis of the results and en face imaging

**Fig. 10:**
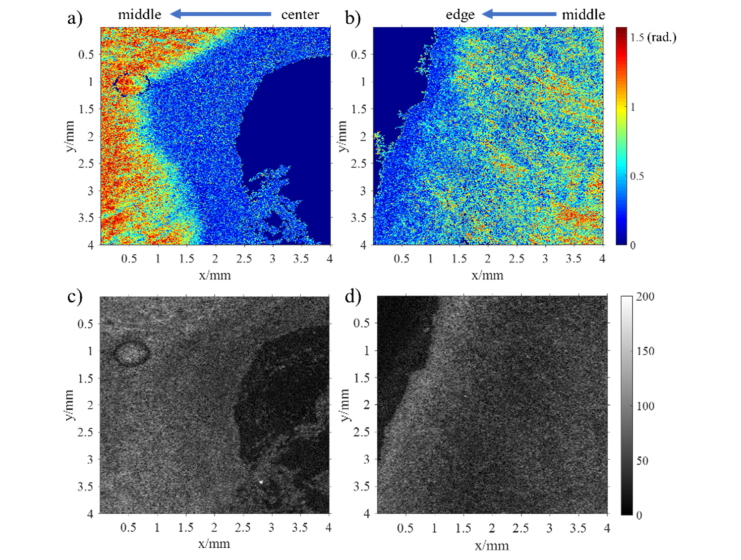
The en-face images of retardance and intensity (at 400 µm depth). a) and b) are the retardance images in center and edge areas respectively. c) and d) are their corresponding intensity images in center and edge areas respectively. 1000 × 952 pixels display the physical size of 4 by 4 mm in the images. Note that the noise has been suppressed by a median filter and a mask in these retardance images, and solid dark blue areas are masked out due to poor SNR.

Twenty non-gravid cervixes were imaged with PS-OCT. The null hypothesis that all regions within the cervix would display a similar mean apparent birefringence was rejected (p<0.05). Post-hoc comparisons using the Bonferroni test indicated significantly higher values for the middle area when compared to the edge or the center regions with an effect size of 33.4%. Two en-face retardance images of cervix from the center and the edge regions respectively (at around 0.4 mm depth) have been included in Fig. 10

as an example. In Fig. 10(a), the en-face retardance image in the center region has a distinct boundary between the center and middle areas. In contrast, in Fig. 10(b), no sharp boundary can be found between the middle and the edge regions. Therefore, PS-OCT is capable of discriminating the boundary of different cervical areas, but the boundary between middle and edge areas could be vague perhaps because of dispersion, arbitrary orientation or degradation of collagen.

**Fig. 11:**
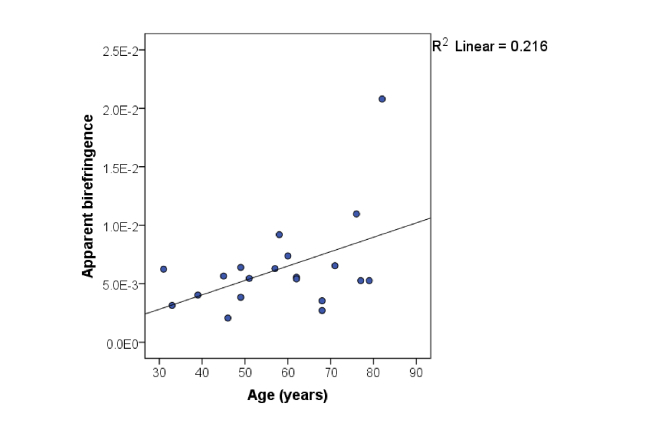
Averaged apparent birefringence of middle area as function of age.

When the apparent birefringence was assessed against the age of the participants using a 2-tailed Pearson’s correlation, a statistically significant difference was seen only for the middle region with a p-value of 0.039, whereas for the central and edge area the correlations were not significant (p = 0.089 and p = 0.625 respectively). In the middle region, apparent birefringence seemed to significantly increase with age, shown in Fig. 11

, which suggests a re-arrangement of collagen fibers after post-menopause consistent with our previous findings employing SHG [47].

No other statistically significant differences were found between birefringence and (1) parity, (2) previous mode of delivery, (3) BMI, (4) indication for surgery, or (5) type of surgery. However, as our study was exploratory in nature and not particularly powered for any of these outcomes, further research is needed before a firm conclusion can be reached between optical and physiological variation in the non-gravid human cervix.

## 4. Conclusion

To the best of our knowledge, this is the first study to ever report on the phase retardance, the birefringence, the orientation of c-axis and depolarization of collagen fibers in human non-gravid cervix using PS-OCT. We have been able to show some of the unique advantages of using PS-OCT to study the cervix, including its ability to (1) easily identify the cervical epithelium and measure epithelial thickness, (2) rapidly image the distribution of cervical collagen, (3) accurately determine birefringence, (4) estimate the 3D alignment of collagen fibers and (5) measure the distinctive depolarization of the cervical tissue. After interrogating 20 cervical cross-sections from non-gravid women using PS-OCT, we found a significant higher birefringence in the middle area compared with the center and edge regions (p< 0.05). As previously seen in studies with SHG, we also identified a significant increase in the apparent birefringence of the middle area with age which could respond to a physiological re-modelling in the cervical collagen fibers as the reproductive function of the cervix diminishes. All in all, we have shown that PS-OCT is capable of assessing the arrangement of cervical collagen objectively and accurately, thus holding promise as a potential tool to better understand cervical remodeling prior to birth. This in turn could lead to earlier identification, more timely prevention and better stratification of management of PTB pending the development of a hand-held probe.
